# Needs of Family Caregivers of People with Lower Limb Amputations: A Scoping Review

**DOI:** 10.3390/bs14040326

**Published:** 2024-04-15

**Authors:** Diana Rodrigues, Rosa Silva, Sofia Castanheira, Luís Carvalho, Cristina Pinto

**Affiliations:** 1 Center for Health Technology and Services Research, Rua Dr. Plácido da Costa, 4200-450 Porto, Portugal; rosasilva@esenf.pt (R.S.);; 2 ICBAS—Abel Salazar Institute of Biomedical Sciences, University of Porto, 4050-313 Porto, Portugal; 3 Nursing School of Porto, 4200-072 Porto, Portugal

**Keywords:** amputation, family caregiver, lower limb, needs assessment

## Abstract

Lower limb amputation affects several parameters of a patient’s life. Family caregivers providing care for these patients experience multiple feelings and needs; knowing caregivers’ needs is essential to prepare them for this new role, as well as the health planning of this type of care. This scoping review aimed to identify and map the needs of family caregivers of people with lower limb amputations. This scoping review was conducted in accordance with the JBI methodological framework and the PRISMA-ScR reporting guidelines. A bibliographical search was carried out on the needs of family caregivers of lower limb amputees in 15 databases. Two independent reviewers extracted data using a data extraction tool developed for this scoping review. Eight studies were included in the present review (n = 6 quantitative studies; n = 2 reviews). Results indicate that family caregivers of people with lower limb amputations may experience an extensive range of needs, as follows: (i) mental health and psychological support, (ii) physical health, (iii) health and well-being, (iv) supportive care, (v) social support, and (vi) educational/informational support. The needs identified in this review can help to develop interventions and programs that provide better support during the situational transition process.

## 1. Introduction

Lower limb amputation (LLA) is a common cause of disability and has a significant global impact on the morbidity of amputees [1,2]. The principal etiological factor is diabetes mellitus, followed by peripheral vascular disease, tumors, orthopedic anomalies, and postoperative embolism [2]. LLA is responsible for physical limitations that can induce restrictions in the functional abilities in the activities of daily living (ADL) of amputees, which can become debilitating via the loss of their independence. These individuals fundamentally face a functional problem, with the restriction of independent movements in their daily routine, leading to increased dependence [2].

LLA is responsible for permanent disability and induces significant alterations in an individual’s life and functioning. It can cause physical, psychological, and emotional dysfunction, requiring caregiver support for general health care and rehabilitation [3,4,5]. Caring for a person with an amputation has been associated with psychological, physical, and mental stress, as well as financial burden on the family, according to several studies, and also has an impact on social relationships. Following the study conducted by Çamur et al. [6], the burden on caregivers is markedly amplified according to the type of amputation; for example, in cases of people with major amputations compared to minor ones, it seems to lead to a greater burden. Within the healthcare framework, it is imperative to acknowledge the substantial load borne by caregivers and address their often-unmet needs [6].

Informal care can be defined as providing unpaid care for a person in need; caregivers are individuals who provide care to family members, spouses, friends, or neighbors, who are older or dependent individuals who need assistance or personal care [7,8]. Informal caregivers can be also referred as “caregivers” or “family caregivers”, most of them are not formally trained in healthcare and are driven by an obligation and social and moral values [8,9,10]. Caregivers are suppliers of physical assistance, emotional support, financial assistance, and other types of care that can lead to stress, fatigue, or physical strain [7,11].

In the process of caring for a dependent person, it is essential to identify the needs of caregivers, taking into account their individual needs and the needs of the person being cared for [12]. Globally, the number of informal caregivers is already large, with the number of people assuming the caregiver role set to increase in the coming years, according to recent research [13]. Informal caregivers provide essential support to their relatives, including assistance in ADLs, provision of medical/nursing tasks, psychosocial support, and communication with healthcare professionals [14]. The impact of caring affects informal caregivers’ health, but the effect differs in consonance with the needs and expectations of caregivers and the available support policies [15,16].

Caregivers’ needs differ depending on the transition phase in which they and the dependent person(s) are found; therefore, a needs assessment should include evaluation in all areas of care, including family relationships, physical and emotional self-care, skills, and knowledge [17]. On the other hand, the person with an amputation has specific needs and, in this sense, identifying the needs and competencies of informal caregivers to enable them to care for their relatives before home discharge can decrease the caregiving burden [18]. Support for informal caregivers is crucial not only for them, but also at the societal level. From the beginning of caring and throughout the caring experience, it is important to meet their needs [19].

Thus, formal support for informal caregivers is vital in maintaining the caregiving network, with the quality-of-care contingent on the effective communication and well-being of the caregivers [20]. Prior to hospital discharge of the dependent individual, it is crucial to assess the abilities and needs of informal caregivers to empower them and minimize their burden, potentially reducing complications to their health and that of the person in their care, as well as subsequent hospital re-admissions [20].

Nurses play a key role in integrating family caregivers into the health care team, ensuring that their needs and contributions are valued. In various healthcare settings, nurses can be trained to assess social support systems, unmet needs, and risk factors for caregivers, thereby facilitating appropriate referrals and information dissemination [21]. A thorough understanding of family caregivers’ unmet needs is essential for the planning and delivery of these need to people providing caregiver-centered care, aiming to bridge care gaps and enhance care quality [21]. Identifying these needs early in the healthcare process is crucial for nurses, enabling the development of targeted interventions to strengthen caregivers’ pivotal capacity.

This scoping review aims to identify, map, and synthesize the needs of family caregivers of patients with lower limb amputations.

The following research question was formulated to guide this study:

“What are the needs of family caregivers of the person with lower limb amputation?”

## 2. Materials and Methods

This scoping review was conducted according to the JBI guidelines [22,23]. Employing a methodological scoping review framework, we systematically examined each research domain to map the existing concepts, sources, and evidence. In addition, this approach facilitated the identification of potential gaps related to the subject under investigation.

To further reinforce the review’s methodological transparency and rigor, we adopted the framework set forth by the Preferred Reporting Items for Systematic Reviews and Meta-Analyses Extension for Scoping Reviews (PRISMA-ScR) checklist [24]. Moreover, a detailed review protocol was constructed to outline the objectives; specify inclusion criteria based on the Population, Concept, and Context (PCC) framework; and articulate the methods for data extraction [25].

### 2.1. Deviations from the Protocol

Between the previously published protocol [25] and the final review, there were some deviations that need to be highlighted; the authors decided to broaden the participants to better comprehend the population under study. Therefore, in this review, for a better definition, family caregivers aged 18 years and above of people over with LLAs were considered in the inclusion criteria for the study population. The protocol [25] only defined the population as family caregivers of people with LLAs, with inaccurate information. Considering this context, it was also broadened to cover the needs of family caregivers of adults with LLAs, who have been discharged home or are being cared for at home or in the community. The protocol only addressed the needs of family caregivers who were responsible for inpatients being discharged home.

The authors broadened the search strategy from the one in the protocol to amplify the available literature on the research topic; the initial search strategy in the protocol used the query (Caregiver* OR “Care Giver*” OR “Carer*”) AND (“Lower Extremit*” OR “Lower Limb*” OR “Membrum infer*”) AND (“Amputat*”). We expanded the search terms to allow access for those using data generated elsewhere, including the search term “home?”. The refinement of this new search was carried out under the guidance of a librarian tailored to each database.

The authors were able to access three additional databases (DOAJ, MEDLINE, via PubMed, and Open Dissertations), which yielded additional peer-reviewed studies. After the search was broadened and updated, a revised search was performed between 2 and 3 August 2003.

### 2.2. Inclusion Criteria

The inclusion criteria of this scoping review were defined based on the research question and the type of evidence added according to the PCC (Participants, Concept, and Context) strategy [22].

Participants: This scoping review considered all studies that included family caregivers aged over 18 years, who were providing care for adults with LLAs. Family caregivers may consist of family members, neighbors, and friends. Studies that included family caregivers of children with LLAs were excluded.

Concept: This review included all studies reporting on the needs of family caregivers aged over 18 years, who were providing care for adults with LLAs. This scoping review focused on the needs of family caregivers providing care for adults with LLAs and studies that did not address family caregivers’ needs or focused only on the needs of amputees were excluded.

Context: All studies that involved family caregivers aged over 18 years, who were providing care for adults with LLAs, who, in turn, had been discharged home or were being cared for at home or in the community (non-institutionalized), were considered in this review. Studies that included family caregivers of adults with LLAs living in nursing homes and other long-term institutions were excluded from the scoping review.

Types of studies: This scoping review considered quantitative and qualitative studies, such as analytical observational studies (e.g., cross-sectional studies) and descriptive analytic studies (e.g., descriptive cross-sectional studies and descriptive qualitative studies). Systematic reviews, literature reviews, and gray literature (books, theses, dissertations, and guidelines) published in indexed sources were also included. All protocols were excluded from the study.

### 2.3. Search Strategy

The search strategy used was the three-step search proposed by the JBI [22] to identify published and unpublished studies to answer the review question. In the first stage, a search limited to the CINAHL Complete (EBSCO) and MEDLINE Complete (EBSCO) databases was conducted using keywords and indexed terms (MESH and DeCS descriptors) to identify articles on the topic, as described in the published protocol [25]. This was followed by an analysis of the index terms and text words used in the titles and abstracts of relevant articles. The index terms and keywords identified in the initial review results were combined using a full search strategy adapted for all databases included in the search (second stage). A research librarian refined the initial search strategy for use in other electronic databases, including both published and unpublished studies in peer-reviewed databases and gray literature sources. The complete list of search terms and the search strategy adapted for each database (see Appendix I) was published online as supplementary material in Zenodo. In the third (final stage), the reference lists of all selected studies that met the inclusion criteria were consulted and analyzed to identify additional studies to be included in the review. One study that met the inclusion criteria was added to the screened reference lists. In the first database search, which was conducted in August (2023), only English, Portuguese, and Spanish articles were included, without date limitations.

The databases searched were Medline Complete (EBSCOhost), CINAHL Complete (EBSCOhost), Academic Search Complete (EBSCOhost), Mediclatina (EBSCO), Science Direct (EBSCO), Scielo (EBSCO), OpenAIRE (EBSCO), DOAJ (EBSCO), Scopus, Web of Science, Medline (PubMed), and Tripdatabase. Sources of unpublished and gray literature were Repositório Científico de Acesso Aberto de Portugal (RCAAP), Open Dissertations (EBSCO), and Google Scholar.

### 2.4. Study Selection

Following the search, all identified records were collated and uploaded to Rayyan (Qatar Computing Research Institute, Doha, Qatar) [26] and duplicates were removed. Two reviewers (DR and RS) independently screened the titles and abstracts of each record and selected the studies that met the inclusion criteria. The full-text studies were retrieved and uploaded to Rayyan. Prior to the start of the study selection, the research team ensured that all reviewers understood the objective, methodology, and established inclusion and exclusion criteria.

In stage two of the study selection, teams of independent reviewers per study (DR and RS; SC and CP) retrieved and assessed full-text studies, verifying their adequacy with the inclusion criteria. Reasons for the exclusion of full-text sources that did not meet the inclusion criteria were recorded and reported in the scoping review. Any disagreements between the reviewers at each stage of the selection process were resolved through discussion or by a third reviewer (LC). The results of the search and study selection process are reported using the Preferred Reporting Items for Systematic Reviews and Meta-Analyses (PRISMA) flow diagram [27].

### 2.5. Data Extraction

Two independent researchers per study (DR and RS; SC and CP) performed data extraction from the selected studies using a data extraction tool, developed according to the recommendations of the JBI [25]. The selected literature was analyzed according to the following specific details: author, year of publication, country of origin, population, methodology, identified needs, study context, and main conclusions relevant to the objective of this review. Any disagreements between the reviewers were resolved through discussion with a third reviewer (LC). It was not necessary to contact the authors to request additional data.

### 2.6. Data Analyses and Presentation

The extracted data and main findings are presented using a descriptive qualitative content analysis, organized according to the objective and review questions of this review. Inductive content analysis was used to collect and analyze the data, without preconceived categories. Needs reported in the studies included in this review were organized and distributed according to their similarities to create categories, and abstraction was used to reduce and group data. Throughout the process, authors engaged in discussions to ensure consensus was reached on category creation and abstracting.

The findings of this scoping review were categorized and reported into two main categories, as follows: (i) needs related to the family caregiver and (ii) needs related to the family caregiver role. The two main categories were divided into six subcategories of needs of family caregivers of people with LLAs, with the inclusion of three categories in each main category. In the main category, (a) needs related to the family caregiver, three subcategories were included, as follows: (i) mental health and psychological needs, (ii) physical health needs, and (iii) health and well-being needs. Concerning the main category, (b) needs related to the family caregiver role, another three subcategories were included, as follows: (iv) supportive care needs, (v) social support needs, and (vi) educational/informational needs. The extracted data were presented in a diagrammatic and tabular format and the tabulated results were accompanied by a narrative summary.

## 3. Results

### 3.1. Study Inclusion

**Figure 1:**
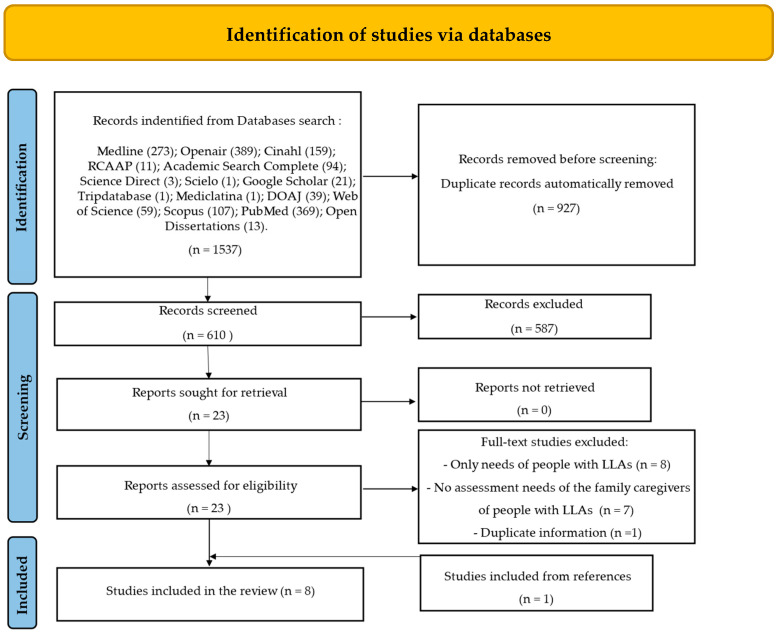
Flow diagram according to PRISMA-ScR guidelines [27].

A total of 1537 bibliographic references were identified through database searches and after the removal of 927 duplicates, 610 records were screened based on title and abstract; 587 were excluded. Using the eligibility criteria for screening titles and abstracts, 23 full-text records were assessed for eligibility and 16 were excluded. The list of ineligible studies following full-text review and principal reasons are presented in Appendix II; the majority of studies were excluded because they focused only on the needs of people with LLAs (n = 8), provided no assessment needs of family caregivers of people with LLAs (n = 7), and provided duplicate information (n = 1). Seven studies that met the inclusion criteria were retained for data extraction and were included in this scoping review. Another study was included from the reference lists of the seven eligible studies, with a total of eight studies included in this review (Figure 1).

### 3.2. Characteristics of Included Studies

The studies included in the review were published between 2009 and 2021 and most of the studies were conducted in Europe (n = 4) [28,29,30,31], with studies also being conducted in Iran (n =1) [32], Brazil (n = 1) [33] and North America (n = 2) [34,35]. Although the inclusion criteria considered studies written in Portuguese, English, and Spanish, seven of the included studies were published in English [28,29,30,31,32,34,35] and one was published in Portuguese [33]. All included studies were published as journal articles and consisted predominantly of studies with a quantitative design (n = 6) [28,29,30,31,32,33] and literature reviews (n = 2) [34,35].

Three of the quantitative studies [28,29,30] have a longitudinal design with the evaluation of the same population of family caregivers of people with LLAs, due to diabetes, using different variables, considering caregiving at three different points of time after amputation, as follows: (i) one month after amputation [28,29,30], (ii) seven months after amputation [28,29,30], and (iii) up to 10 months after amputation [28,29,30]. A total of 110 family caregivers of individuals with LLAs due to diabetes participated in the first evaluation [28,29,30], 101 family caregivers participated in the evaluation seven months after amputation [28,29,30], and 84 family caregivers were included in the final evaluation up to 10 months after amputation [28,29,30]. One study [28] explored the mediating role of caregivers’ traumatic stress related to amputation in the relationship between caregivers’ stress, social support, and help in caregiving, considering the duration of caregiving after amputation. Another study [29] evaluated family caregivers’ use of a model of adaptation to chronic illness and assessed the changes in the lives of family caregivers of people with amputations due to diabetes for up to 10 months after amputation, evaluating the role of continuously experienced stress and the support needs in caregiving. In the third study [30], changes in family caregivers’ burden of caring for a person with a LLA due to diabetes were evaluated at three different points, up to 10 months after amputation, using a model of caregiving burden.

The number of participants in the studies in the review ranged from 14 to 464, apart from two studies in which the sample size could not be determined due to the study methodology. Four studies [28,29,30,33] examined only family caregivers’ perspectives regarding the care of the LLA amputee, including only family caregivers as participants; in two studies [31,32], both caregivers and patients’ perspectives were examined; and in two studies [34,35], only patients’ perspectives regarding family caregivers in the caring process were examined. One of the studies reports on the caregiver–patient relationship in the process of caring for an LLA amputee, considering only wives as family caregivers and participants.

The majority of the studies (n = 4) [28,29,30,33] in this review report on family caregivers of patients with dysvascular amputation that include peripheral arterial disease or diabetes or both; one study reports on patients with dysvascular and traumatic amputation; another study reports on patients with traumatic amputation and their family caregivers [31]; in another study, we had wives (as family caregivers) of patients with LLAs due to war injuries [32]. A range of data collection methods were used in the included studies to conduct research, including surveys, questionnaires, and semi-structured interviews. The characteristics of the studies included in this review are summarized in Table 1.

### 3.3. Summary of Results

**Figure 2:**
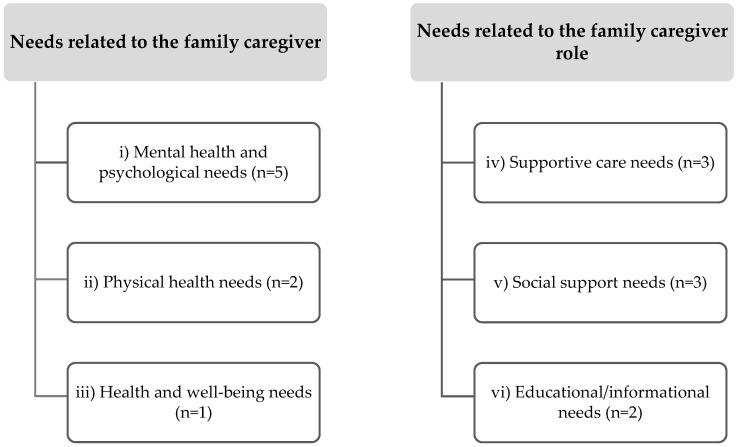
Needs of family caregivers of people with LLAs.

We mapped and summarized the available evidence related to the needs of family caregivers of people with LLAs. From the content analysis to the extracted data, two broad categories emerged, as follows: (i) needs related to the family caregiver and (ii) needs related to the family caregiver role, which were dispersed across six sub-categories. Mental health and psychological needs were identified in six of the included studies, followed by social support needs and supportive care needs, both identified in three studies, being the more relevant identified needs. Physical health and educational/informational needs were identified in two studies and health and well-being needs were referred to in one study each. Figure 2 illustrates the presentation of the categorized needs.

#### 3.3.1. Mental Health and Psychological Support Needs of FCs of People with LLAs

Needs related to mental health and psychological support of the family caregiver of a person with an LLA were more frequently reported in this review, being identified in five studies [28,29,31,32,33]. Amputation is a traumatic event that seems to trigger caregivers’ traumatic stress and caregivers should be screened for stress during the first month after the patient’s amputation. The characteristics of the traumatic event (amputation) affect the mental quality of life (QoL) and burden on caregivers through the traumatic nature of the event lived by the patient [28]. Ganjparvar et al. [32] referees that family caregivers of LL (lower limb) amputees have lower scores in mental health than in the general population; family caregivers of LL amputees with mental health disorders have lower mental health quality than their counterparts, which influences their QoL.

One of the included studies [33] referred to the fact that the psychological support needs of the family caregiver of a person with an LLA, regarding the negative impact of amputation in their lives and the emotional aspect associated with general tension, isolation, disappointment, and environment, is significant. Costa et al. [29] reported that between one and seven months after amputation, family caregivers experience an increase in physical exhaustion and stress associated with caring for a disabled person, which decreases their mental health quality. The same author mentions that caregivers need to cope with caregiving demands and maintain their mental health quality.

Regarding caregiving of the person with an LLA, it was reported that caregivers frequently put amputees’ needs before their own and 80% of the caregivers referred to their need for psychological counseling after hospital discharge [31].

#### 3.3.2. Physical Health Needs of FCs of People with LLAs

Needs related to physical health and physical problems of the family caregiver of a person with an LLA were identified in two studies [29,32]. Providing care for disabled people at home exposes caregivers to a potentially higher risk of physical problems. Caregivers who care for amputees from the beginning of their condition have a lower QoL in the domain of physical functioning, with limited physical activity, than the general population [32]. The study by Costa et al. [29] mention that one and seven months after amputation is a critical period, given the increasing physical symptoms in caregivers associated with the exigency of care for a person with reduced mobility, for caregivers to receive support to help reduce physical symptoms.

#### 3.3.3. Health and Well-Being Needs of FCs of People with LLAs

Ganjparvar et al. [32] found that family caregivers of bilateral LLAs have unfulfilled health and well-being needs and a poor QoL, when compared with the general population. Efforts to enhance caregivers’ QoL and fulfill their health and well-being needs should include training amputees and their caregivers regarding the social, emotional, and psychological aspects of their lives [32].

#### 3.3.4. Supportive Care Needs of Family Caregivers of People with LLAs

Three of the included studies indicated that supportive care needs are important for family caregivers of people with LLAs [28,29,30]. In all three studies, the family caregiver of a person with an LLA was evaluated at three different time points after amputation (1, 7, and 10 months). One of the studies [28] considered the importance of help in caregiving in the first month after amputation, helping to decrease the stress and caregiving burden associated with traumatic stress associated with amputation. Costa et al. [29] evaluated the life changes in family caregivers of LL amputees for up to 10 months, suggesting that one to seven months after surgery, family caregivers experience exhaustion due to increasing demands on care, requiring greater help in caregiving tasks. In the third study [30], family caregivers needed increasing help with demanding caregiving activities and the findings showed that in the caregivers who received care assistance, the caregiving burden decreased over time.

#### 3.3.5. Social Support Needs of FCs of People with LLAs

Considering the duration of caregiving, family caregivers of people with LLAs begin to show signs of stress and exhaustion, one to seven months after amputation, with a negative impact on caregiving, needing social support in this period to help them with caregiving demands [28]. Social support needs were identified in a study by Costa et al. [30], who reported that family caregivers need a support network to help in caregiving tasks, this help is crucial preventing caregiving burden and stress when caring for others [30]. A study by Ganjparvar et al. [32] showed that family caregivers of amputees have a lower social functioning than the general population, with a lack of social support to aid them in dealing with the caregiving burden.

#### 3.3.6. Educational/Informational Needs of FCs of People with LLAs

A gap in communication between healthcare providers, amputees, and caregivers was identified in one study [34], and postoperative care and communication among health providers, patients, and their families are essential in the planning of care and for a successful transition to a new life.

Regarding educational and training skills, Latlief et al. [35] mentioned that family caregivers of amputees need education related to the care of the amputee stump and training in transferring techniques to help the amputee.

## 4. Discussion

To the best of our knowledge, this scoping review is the first to systematically and comprehensively identify the needs of family caregivers of people with LLAs, providing an overview of the existing knowledge, including the characteristics and results of a heterogeneous sample of studies that describe caregivers’ needs.

The mental health and psychological support needs of family caregivers of people with LLAs are reported in this review, as they are the most common needs related to these caregivers. Previous findings indicate that by providing unpaid care to dependent family members or friends, informal care is often associated with mental health effects and caregiving might result in a higher prevalence of depressive feelings and a lower mental health score [7]. Caregivers can experience irritability, anxiety, and other negative emotions due to their heavy care burden and pressure; they need psychological consultations to ease the pressure and to help maintain a good emotional state [7]. Caregivers need skills training and psychological support, and systematic intuitional services should be tested to support caregivers and improve educational outcomes and the QoL for caregivers and patients, especially for those with high psychological needs [36].

Supportive care needs are considered important for the family caregiver of a person with an LLA, as having support in caregiving can help them to deal with the demands of caring for a disabled person and help them manage their caregiving burden over time. Caregivers are responsible for specific tasks as part of the support they provide to their care recipients, which can be categorized into activities of daily living (ADLs) and instrumental activities of daily living (IADLs). In ADLs, caregivers provide support for getting in and out of chairs and beds, using the toilet, bathing, personal care, dealing with incontinence/diapers, and feeding. IADLs include managing finances, grocery and shopping, preparing meals, housework, transportation, and performing nursing/medical tasks [37]. Most caregivers provide support in multiple ADLs and IADLs, emotional support to the care recipient, and communication with healthcare providers. Caregivers reported unmet support–care needs in several areas. Care recipients’ well-being and outcomes are affected by caregivers’ needs, demonstrating that it is crucial to address the unmet needs of caregivers in all five supportive care areas (medical/nursing training, help accessing services, respite care, support groups, and caregiver counseling) [14]. Caregivers’ assessments should comprise their ability to perform required tasks and the types of support and training they might need to perform their roles [38].

Findings in this review show that family caregivers need social networks that include family, community, or health professionals to help them in caregiving tasks, giving them social support that can contribute to preventing an increase in caregiving burden. Social support can be referred to as family, friends, neighbors, and other community members, who can provide psychological, physical, financial, or other support in times of need [39]. Being a caregiver can be very demanding; caregiving tasks that include bathing, feeding, bedding, and changing care recipients’ clothes can be physically demanding and caregivers’ and families’ lives can be very restricted [40,41]. Caregiving can have a damaging effect on caregivers’ lives; they have a significant need for social support to help them handle caregiving responsibilities and tasks [41]. The level of social support that caregivers have to satisfy their emotional, informational, and practical needs of caring can impact their well-being and maintain their health balance [42]. Caregivers need reasonable social support from both family and the community to enable them to participate in social activities and to reduce the stress and burden on caregivers [40].

Caring for a person with a physical disability, such as an amputee, can be very demanding for family caregivers, most of the time resulting in chronic stress and physical exhaustion, affecting physical and physiological well-being. Most family caregivers of people with an LLA have physical health needs associated with exhaustion that affect their QoL, and also affects their health and well-being. Providing care to family members, friends, spouses, or neighbors can be very demanding and can lead to physical strain, fatigue, or stress, with a negative impact on the physical health of informal caregivers [7]. The findings suggest that caregiver health and well-being are influenced by more than the burden of care, despite the burden being clearly associated with QoL [43]. Target interventions to support caregivers, reduce their health effects, and enhance their well-being are needed [7,43].

Postoperative care is an important part of the transition of a person with an LLA and their family caregiver in returning home, and a discharge plan with information, training, and communication is essential for the patient and family. In the studies of this review, family caregivers’ needs were related to education and information related to the care of the person with an LLA at home. When caregivers acquire more knowledge about aspects of care, their performance improves and educational interventions enhance their knowledge, attitudes, and practices [44]. Nurses should consider informational support as an important nursing intervention during hospitalization [45]. After informational needs have been met, caregivers should benefit from training in problem-solving skills [46]. During care of the recipient and healthcare provider encounters, assessments of the caregiver should be made to inform decisions regarding whether the caregiver is capable of assuming a role and the types of training required [38]. Caregiver confidence and the ability to manage daily care challenges can be improved through education and skill training [38].

This scoping review underscores the importance of adapting clinical practice to better support family caregivers of individuals with an LLA, as it draws attention to their unmet needs. The development of a structured program for training and supporting informal caregivers’ skills can have an impact on caregiving burden levels and can improve their global health condition [47]. As the disease progresses, the caregivers’ needs decrease. Engaging with community support resources can lighten the caregiving load and fulfill the diverse requirements of patient care [48]. Assessing caregivers’ needs can determine the development of support services, provide better care and mental health, and help decrease caregiving burden.

### 4.1. Limitations

A standard protocol was followed in this scoping review with an extensive literature search using several databases, although some relevant studies may not have been included. Another limitation of this review is the inclusion of only articles in three languages, Portuguese, English, and Spanish (proficiency domain of the researchers); therefore, some relevant studies may not have been included.

A small number of studies that referred to the needs of family caregivers of people with LLAs were included in this review. The studies did not focus only on the study and identification of caregivers’ needs. There is insufficient information relating to the needs of the family caregivers of people with LLAs, which can reveal a gap in the knowledge and studies regarding the unmet needs of family caregivers of people with LLAs, when discharged home.

### 4.2. Implications for Research and Practice

This scoping review provides insight into the needs related to the family caregivers of people with LLAs, which could be considered as a basis for the development of future studies. In this field, it is important to conduct studies to understand the changes in the needs of the family caregiver of a person with an LLA through the disease. Studies should be carried out to understand the impact of the patient’s condition and the type and severity of the amputation on the needs of family caregivers. These studies can help inform the design of changes in formal care.

This scoping review can contribute to the development of empirical studies that demonstrate the importance of identifying caregivers’ needs regarding the burden of care. Understanding and assessing these needs are fundamental for clinical practice and to help improve the therapeutic relationship between patients, caregivers, and health professionals. Our findings point to the importance of changes in health practices concerning the family caregivers of people with LLAs, as health services should provide access to resources and support to address caregivers’ needs.

## 5. Conclusions

The results provide an overview of the literature on the needs of family caregivers of people with LLAs, which provides insights into the limited literature in this area. Family caregivers are an important part of the care network of people with LLAs; therefore, as care providers, they require support. Assessing family caregivers’ needs before home discharge can help to design a care plan to empower them to care for their relatives, thereby decreasing the caregiving burden and hospital admissions.

Understanding caregivers’ needs may enable future interventions and educational programs to address the needs of LLA caregivers, providing them with better support in the transition to their caregiving role. Nurses should acknowledge the community resources and social networks of caregivers to continue their follow-up and identify the unmet needs of the family caregiver of a person with an LLA during the care process. This can help in the design of a care plan adjusted to the caregiver’s capacities and needs and can reduce the negative consequences of family care.

## Figures and Tables

**Table 1:** Summary of included studies.

Author(s) (Year), Country	Methodology	Participants Study Context	Identified Needs	Main Conclusions
Costa et al. (2021) [28], Portugal	Longitudinal Study (Quantitative)	110 family caregivers of people with diabetic foot amputations (1 month after amputation); 101 family caregivers (7 months after amputation); 84 family caregivers (up to 10 months after amputation); Living at home	(i) Mental health and psychological needs (iv) Supportive care needs (v) Social support needs	Family caregivers show signs of stress during the first month after patient’s amputation. Traumatic stress mediates the relationship between caregiver stress and mental QoL, emphasizing the importance of mental health in the family caregiver. Family caregivers need help in caregiving, especially 1 month after amputation, because of the increase in caregiving burden. Between 1 and 7 months after amputation, family caregivers show signs of traumatic stress with negative consequences in caregiving, needing social support in caregiving to help them with the increase traumatic stress in this period.
Costa et al. (2020) [29], Portugal	Longitudinal Study (Quantitative)	110 family caregivers of people with diabetic foot amputations (1 month after amputation); 101 family caregivers (7 months after amputation); 84 family caregivers (up to 10 months after amputation); Living at home	(i) Mental health and psychological needs (ii) Physical health needs (iv) Supportive care needs	Between 1 and 7 months after surgery appears to be a critical period, given the increase in physical symptoms; caregivers who presented high levels of physical symptomatology showed low levels of mental QoL over time. Seven months after the amputation, caregivers experience a greater number of physical symptoms, due to the strain of caring for patients with reduced mobility. In the period between 1 and 7 months after amputation, an increase occurs in the physical symptomatology of the family caregivers due to exhaustion, needing support in caregiving tasks to help deal with physical exhaustion.
Costa et al. (2018) [30], Portugal	Longitudinal Study (Quantitative)	110 family caregivers of people with diabetic foot amputations (1 month after amputation); 101 family caregivers (7 months after amputation); 84 family caregivers (up to 10 months after amputation); Living at home	(iv) Supportive care needs (v) Social support needs	Family caregivers need increasing help with caregiving activities up to 10 months after amputation, to prevent the burden and stress of care from increasing over time. Caregiver burden increases in the first seven months after amputation and family or social support are needed to prevent the development of caregiving burden and other symptoms.
Tsoulou et al. (2019) [31], Grece	Cross-sectional Study (Quantitative)	50 hospitalized patients who had undergone traumatic amputations (lower limb, fingers) and 50 family caregivers; Hospital discharge/returning home	(i) Mental health and psychological needs	Caregivers frequently put others’ needs before their own and sacrifice leisure time. Patients’ and caregivers’ psychological state is of great importance for effective treatment. After hospital discharge, 80% of the caregivers were referred for psychological counseling.
Ganjparvar et al. (2016) [32], Iran	Cross-sectional Study (Quantitative)	232 amputees with LLA due to different injuries during war and 232 family caregivers (wives); Living at home	(i) Mental health and psychological needs (ii) Physical health needs (iii) Health and well-being needs (v) social support needs	Caregivers need to assess care and service to maintain physical and mental health and help to promote their QoL. Caregivers responsible for the care of the person with amputations from the beginning of their condition have a lower quality of life (QoL) in the domain of physical functioning with limitations in physical activity. Caregivers of the person with bilateral LLAs have unfulfilled health and well-being needs and a poor QoL. Caregivers have lower scores in the domain of general health when compared with the general population. Compared with the general population, family caregivers of people with LLAs have lower scores in social function and mental health. Social support helps to mitigate the effect of caregivers’ burden.
Foss et al. (2009) [33], Brazil	Descriptive Cross-sectional Study (Quantitative)	87 family caregivers of people with dysvascular LLAs; Living at home	(i) Mental health and psychological needs	The emotional aspect of LLA caregivers associated with general tension, isolation, disappointment, and environment is significant. LLAs’ caregivers need guidance and psychological support.
Bennett, J. (2016) [34], USA	Literature Review	Amputees with LLAs due to diabetes; Hospital discharge/returning home	(vi) Educational/Informational needs	Communication is essential for consistency in the planning of care for the patient and their caregivers. Postoperative care along with communication among health providers, patients, and their families are essential for a successful transition to a new life.
Latlief et al. (2012) [35], USA	Literature Review	Amputees with LLAs due to dysvascular conditions or traumatic events; Returning home	(vi) Educational/Informational needs	There is a greater risk of injury in family caregivers of the person with amputation, transferring into/out of chairs, cars, beds, bathtubs, and other environments, given that most of them are untrained. Families of the person with a LLA need to be educated on issues such as skin monitoring, stump sock management, donning and doffing prosthesis, residual limb hygiene, and componentry inspection and maintenance.

Legend: QoL, quality of life; LLA, lower limb amputation; USA, United States of America.

## Data Availability

No new data were created or analyzed in this study. Data sharing was not applicable in this study.

## Supplementary Materials

**Appendix I—Search strategy:** Detailed information on the search strategy developed and refined according to each database included in the review can be found in the following link: https://doi.org/10.5281/zenodo.10793574; **Appendix II—Studies Ineligible Following Full-Text Review:** Listing of all the ineligible studies following a full-text review, specifying the reasons for exclusion for each of them, can be found in the following link: https://doi.org/10.5281/zenodo.10974673. References [49,50,51,52,53,54,55,56,57,58,59,60,61] are cited in the supplementary materials.
